# Induction of Peroxisomes by Butyrate-Producing Probiotics

**DOI:** 10.1371/journal.pone.0117851

**Published:** 2015-02-06

**Authors:** Huachun Weng, Kosuke Endo, Jiawei Li, Naoko Kito, Naoharu Iwai

**Affiliations:** Department of Genomic Medicine, Research Institute, National Cerebral and Cardiovascular Center, Suita, Osaka, Japan; Clermont Université, FRANCE

## Abstract

We previously found that peroxisomal biogenesis factor 11a (*Pex11a*) deficiency is associated with a reduction in peroxisome abundance and impaired fatty acid metabolism in hepatocytes, and results in steatosis. In the present study, we investigated whether butyrate induces *Pex11a* expression and peroxisome proliferation, and studied its effect on lipid metabolism. C57BL/6 mice fed standard chow or a high-fat diet (HFD) were treated with tributyrin, 4-phelybutyrate acid (4-PBA), or the butyrate-producing probiotics (*Clostridium butyricum* MIYAIRI 588 [CBM]) plus inulin (dietary fiber), and the body weight, white adipose tissue, serum triglycerides, mRNA expression, and peroxisome abundance were evaluated. Tributyrin or 4-PBA treatment significantly decreased body weight and increased hepatic mRNA expression of peroxisome proliferator-activated receptor-α (*PPARα*) and *Pex11a*. In addition, 4-PBA treatment increased peroxisome abundance and the expression of genes involved in peroxisomal fatty acid β-oxidation (acyl-coenzyme A oxidase 1 and hydroxysteroid [17-beta] dehydrogenase 4). CBM and inulin administration reduced adipose tissue mass and serum triglycerides, induced *Pex11a*, acyl-coenzyme A oxidase 1, and hydroxysteroid (17-beta) dehydrogenase 4 genes, and increased peroxisome abundance in mice fed standard chow or an HFD. In conclusion, elevation of butyrate availability (directly through administration of butyrate or indirectly via administration of butyrate-producing probiotics plus fiber) induces *PPARα* and *Pex11a* and the genes involved in peroxisomal fatty acid β-oxidation, increases peroxisome abundance, and improves lipid metabolism. These results may provide a new therapeutic strategy against hyperlipidemia and obesity.

## Introduction

Peroxisomes are ubiquitous organelles bounded by a single membrane. Mammalian peroxisomes have many important metabolic functions, including β-oxidation of very long-chain fatty acids, α- and β-oxidation of long branched-chain fatty acids, synthesis of cholesterol and ether lipids, and H_2_O_2_ metabolism [1–3]. They are highly versatile and dynamic organelles whose size, shape, number, and protein content adapt to various cell types, metabolic requirements, and extracellular stimuli [3–6]. We recently reported that a reduced abundance of functional peroxisomes is associated with fatty liver and aggravated interstitial lesions in the kidney through phenotypic analyses in peroxisomal biogenesis factor 11a (*Pex11a*) knockout mice [4, 7]. Fenofibrate appeared to alleviate fatty liver and interstitial lesions in the kidney by increasing peroxisome abundance [4, 7].

Butyrate is another candidate *in vivo* peroxisome proliferator [8]. Butyrate is a short-chain fatty acid (SCFA) produced by microbiota in the colon and distal small intestine by fermentation of resistant starch, dietary fiber, and low-digestible polysaccharides [9].

*Clostridium butyricum* MIYAIRI 588 (CBM) is a butyric acid-producing gram-positive anaerobe found in soil and the intestines of humans and other animals [10]. Inulin belongs to a class of dietary fibers known as fructans, which are used in the food industry and are considered functional food ingredients. Experimental studies have shown that their use reduces the synthesis of triglycerides and fatty acids in the liver and decreases the levels of these compounds in serum [11, 12]. Furthermore, several studies have shown that CBM and dietary fiber improve high-fat diet (HFD)-induced nonalcoholic fatty liver disease [11–15]. It is likely that these observed effects might be due to increased synthesis of butyrate in the colon, which increases the abundance of peroxisomes in the liver. The aim of the present study was to examine whether butyrate or CBM and dietary fiber induce peroxisome proliferation in the liver and improve lipid metabolism in mice.

## Materials and Methods

### Experimental animals and diet

Male C57BL/6 mice were obtained from SLC Japan (Shizuoka, Japan) and divided into two groups matched for body weight: vehicle and treatment. The mice were housed in a temperature-controlled pathogen-free room with light from 07:00 to19:00 h (daytime) and had free access to food and water. All experimental protocols involving mice were approved by the Ethical Review Board of the National Cerebral and Cardiovascular Center (Japan) (Permit Number: 14015) and performed according to the Guidelines for the Care and use of Experimental Animals of the National Cerebral and Cardiovascular Center and the National Institutes of Health’s Guide for the Care and Use of Laboratory Animals. Experiments were conducted such that pain and discomfort to the mice were minimized. Normal chow and HFD were obtained from Oriental Yeast (Tokyo, Japan). Normal chow and water, supplemented with 1% 4-phenolbutyrate acid (4-PBA, Sigma-Aldrich; St. Louis, MO, USA), were given to the animals for 2 weeks. Mice fed normal chow were injected intraperitoneally with 0.5 mg/g tributyrin or vehicle (glycerol) once. Normal chow or HFD supplemented with 3% CBM (wt/wt, Miyarisan Pharmaceutical Co.; Nagano, Japan) and/or drinking water containing 1% inulin (Nippon Garlic Co.; Gunma, Japan) were given to the animals for 2 weeks. The percentage increase in body weight (BW) was calculated as follows: (BW at week W/ BW at week 1) × 100.

### Metabolite assays

Mice were subsequently euthanized under pentobarbital sodium anesthesia. Blood was collected from the abdominal vein and centrifuged for 10 min to collect sera. Sera were kept at −80°C. Serum triglyceride was measured using DRI-CHEM 7000 (Fujifilm; Tokyo, Japan).

### Immunofluorescence microscopy and peroxisome abundance

Liver tissue was fixed in Bouin’s fixative solution (Bioscience; Allentown, PA, USA) overnight at 4°C and then placed in 70% alcohol. Livers were washed using phosphate-buffered saline (PBS), embedded in optimal cutting temperature compound, and then sectioned at 7 μm. Sections were washed twice in PBS and permeabilized for 10 min in PBS containing 0.5% Triton X-100. The sections were again washed twice with PBS and then incubated with blocking buffer (2% bovine serum albumin, 0.5% Triton X-100 in PBS) for 60 min, followed by incubation with rabbit antibody against peroxisome membrane protein 70 (anti-PMP70: S34201, 1:1000, Invitrogen; Carlsbad, CA, USA) overnight at 4°C. Sections were subsequently washed five times with PBS containing 0.5% Triton X-100 and incubated with Texas Alexa Fluor 488-conjugated goat anti-rabbit secondary antibodies (A-11008, 1:1000, Invitrogen) for 60 min at room temperature. Sections were washed five times with PBS and mounted on glass slides. To determine the number of peroxisomes in liver sections, fluorescence images were acquired using a confocal laser-scanning microscope (Olympus; Tokyo, Japan) and analyzed using Image J software (National Institutes of Health; Bethesda, MD, USA). At least 20 randomly selected independent fields were photographed. Peroxisomes were counted using the Particle Analysis package of Image J.

### Gene expression analysis

RNA was extracted from livers using Trizol reagent (Invitrogen) according to the manufacturer’s instructions. cDNA obtained by reverse transcription was amplified using the appropriate primers, and mRNA expression levels were determined by real-time reverse transcription-polymerase chain reaction using a commercial kit (Applied Biosystems; Foster City, CA, USA). All samples were analyzed in triplicate multiplex reactions, and both the levels of the gene of interest and β-actin, as an internal control, were measured, as described previously [4].

### Quantification and statistical analysis

Data are presented as the mean ± SD. Statistical analyses were performed using one-way analyses of variance, a multivariate analysis of variance, and then with Student’s *t*-tests using the JMP statistical analysis package (SAS Institute; Cary, NC, USA). *P* < 0.05 was considered significant.

## Results

**Fig 1:**
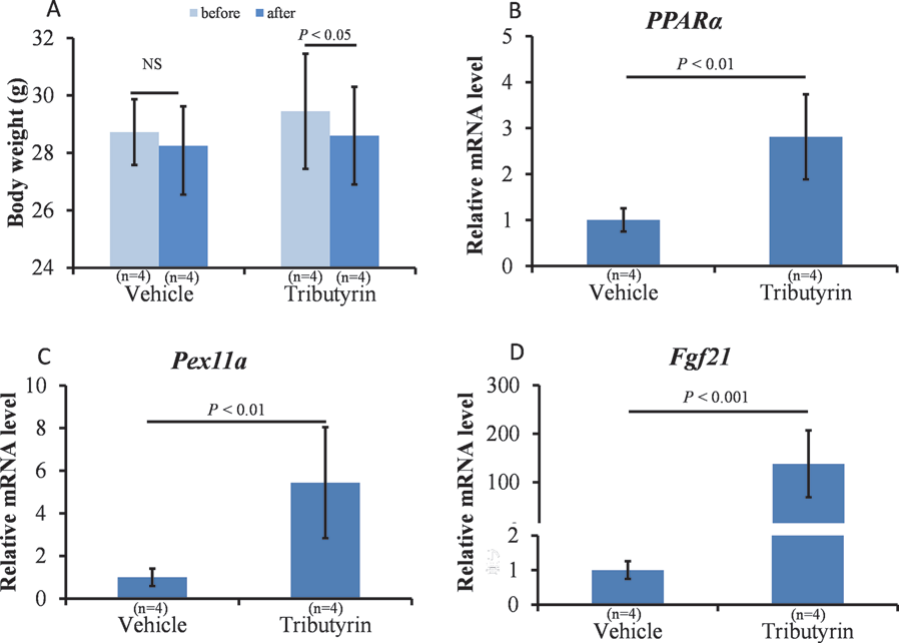
Effects of tributyrin on body weight (BW) and mRNA expression in the liver. C57BL/6 mice fed a normal diet were injected intraperitoneally with tributyrin (0.5 g/kg BW) or vehicle (glycerol) once. BW was measured and mice were sacrificed after 6 h. mRNA levels of the indicated genes in the livers were measured using real-time reverse transcription-polymerase chain reaction. β-actin was used as an internal control. Bars represent the mean ± SD. NS: Not significant.

Tributyrin is a triglyceride containing three butyrate moieties; it has low toxicity and is rapidly absorbed and hydrolyzed to butyrate by plasmatic esterases. Tributyrin treatment significantly reduced BW at 6 h after injection (Fig. 1A, *P* < 0.05), probably due to reduced water and food intake. Tributyrin treatment significantly increased peroxisome proliferator-activated receptor-α (*PPARα*) and *Pex11a* mRNA expression (Fig. 1B-C, *P* < 0.01). Intriguingly, tributyrin treatment markedly increased the expression levels of fibroblast growth factor 21 (*Fgf21*), which plays a critical role in maintaining energy homeostasis [16], by approximately 150-fold (Fig. 1D, *P* < 0.001).

**Fig 2:**
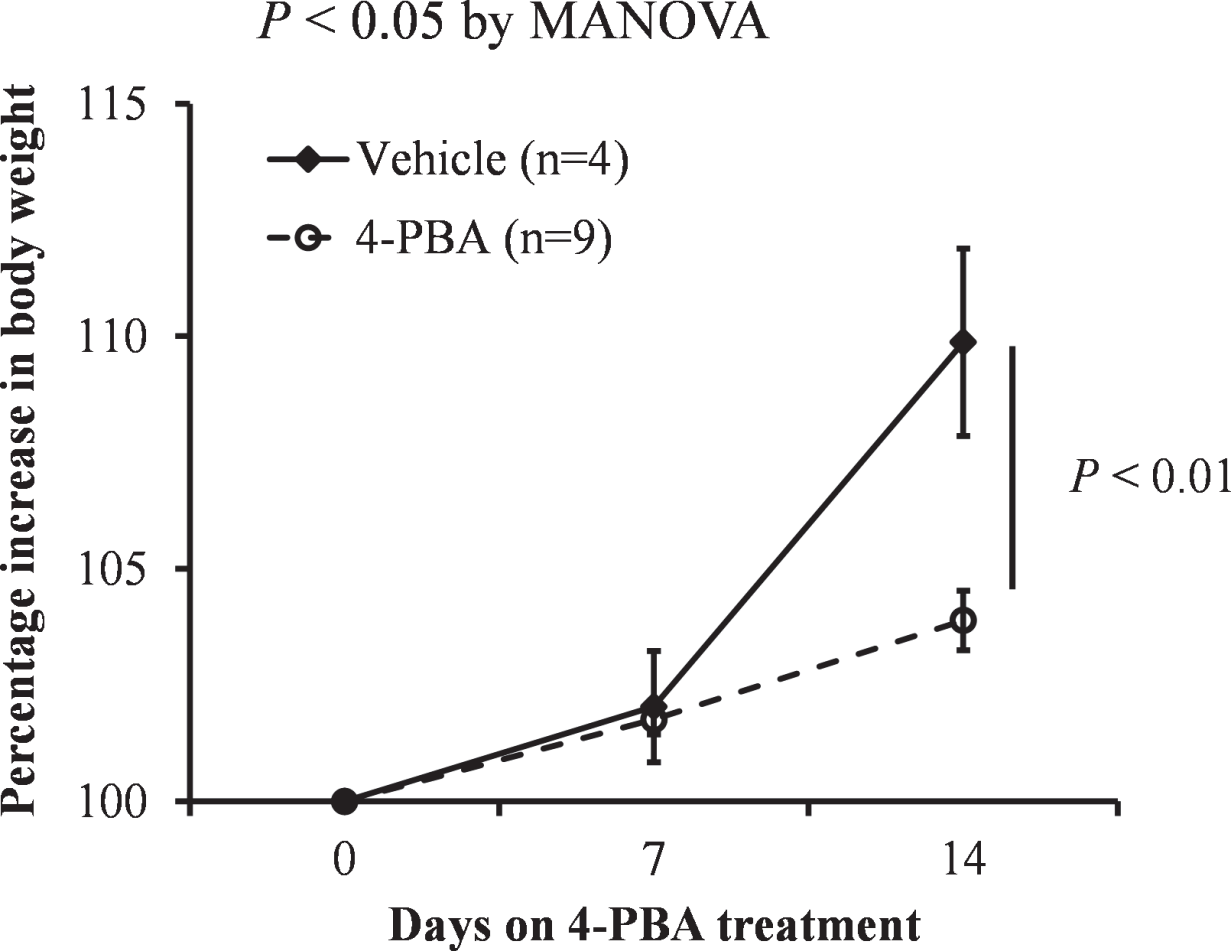
Effects of 4-phenolbuturate acid (4-PBA) on body weight (BW). C57BL/6 mice were fed a normal diet and drinking water supplemented with 1% 4-PBA (wt/wt) for 2 weeks. BW was measured weekly. The percentage increase in BW was calculated as: (BW at week W/ BW at week 1) × 100. Bars represent the mean ± SD. MANOVA: multivariate analysis of variance.

**Fig 3:**
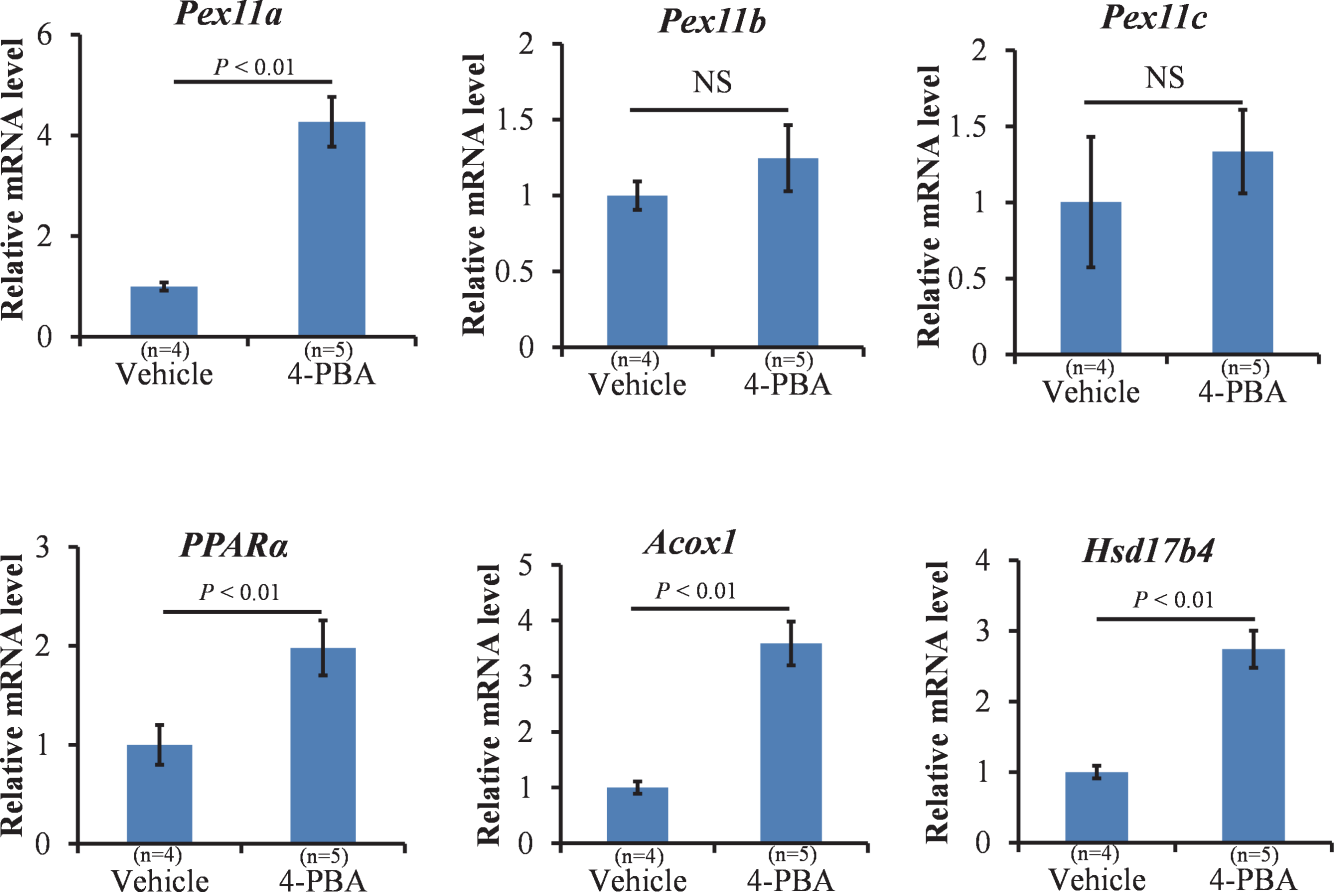
Effects of 4-phenolbuturate acid (4-PBA) on mRNA expression in the liver. C57BL/6 mice were fed a normal diet and drinking water supplemented with 1% 4-PBA (wt/wt) for 2 weeks. mRNA levels of the indicated genes in the livers were measured using real-time reverse transcription-polymerase chain reaction. β-actin was used as an internal control. Bars represent the mean ± SD. NS: Not significant.

**Fig 4:**
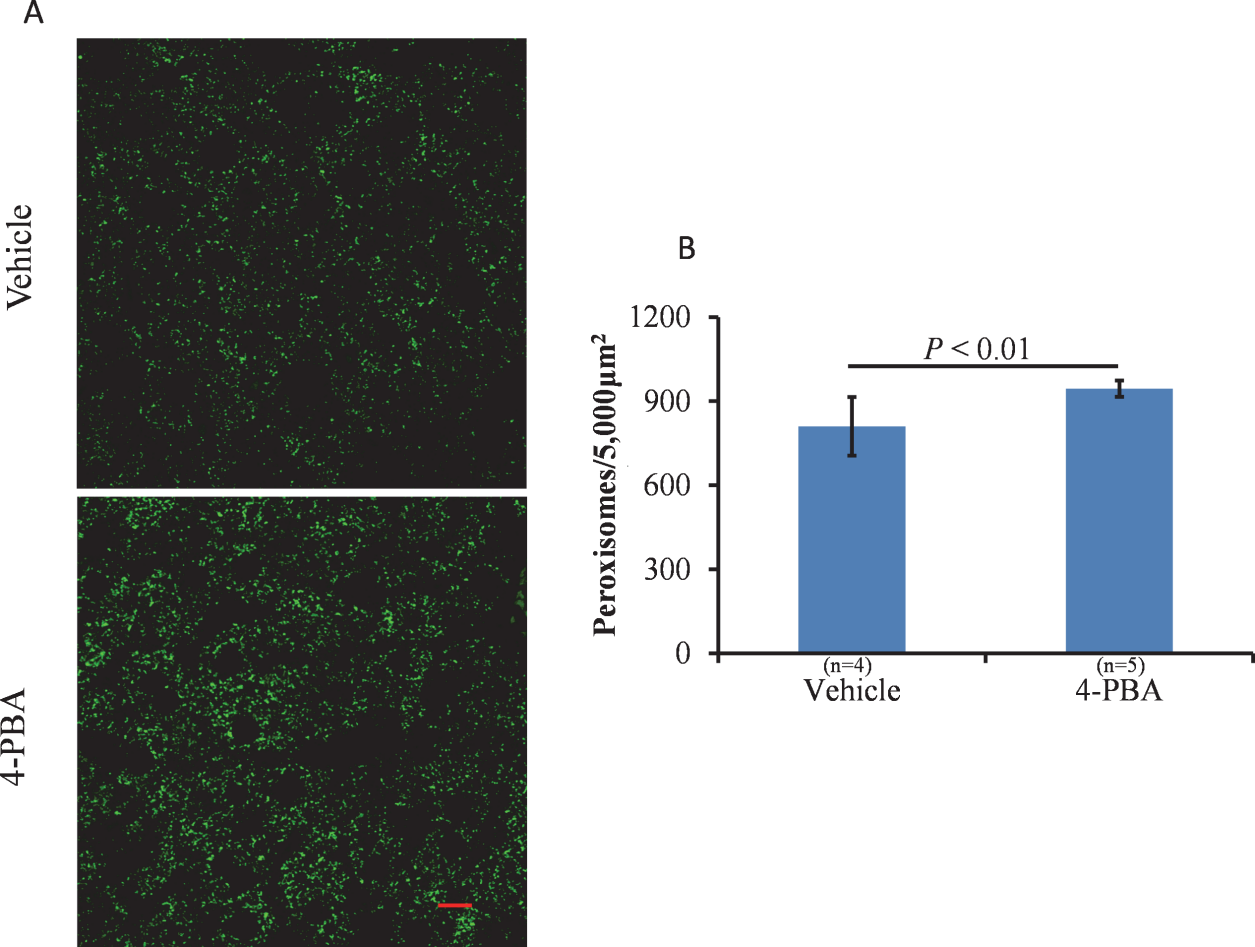
Effects of 4-phenolbuturate acid (4-PBA) on peroxisome abundance in the liver. **A**, C57BL/6 mice were fed a normal diet and drinking water supplemented with 1% 4-PBA (wt/wt) for 2 weeks. The liver sections were examined using immunofluorescence staining with an antibody to peroxisome membrane protein 70 (Alexa Fluor 488, green channel). Scale bar: 5 μm. The fluorescence emitted by Alexa Fluor 488 was visualized using a confocal laser-scanning microscope. **B**, Peroxisomes were counted using the Particle Analysis package of Image J. Bars represent the mean ± SD.

To eliminate the disadvantage of the short half-life of butyrate, normal chow and water supplemented with 1% 4-PBA (wt/wt) were given to the mice for 2 weeks. The percentage increase in BW was significantly reduced after 2 weeks of 4-PBA treatment (Fig. 2, *P* < 0.01). *PPARα* and *Pex11a* mRNA levels in the livers of the treated group were significantly higher than those of the control group (Fig. 3).The expression of genes involved in peroxisomal fatty acid oxidation (acyl-coenzyme A oxidase 1 [*Acox1*] and hydroxysteroid [17-beta] dehydrogenase 4 [*Hsd17b4*]) was induced by 4-PBA treatment. However, mRNA expression of the other *Pex11* genes (*Pex11b* and *Pex11c*) was not induced by 4-PBA treatment. Furthermore, as shown in Fig. 4A, the peroxisomes of the livers were stained with PMP70. The number of peroxisomes in the livers of the treated group was significantly higher than that in the livers of the control group (Fig. 4B, *P* < 0.01).

**Fig 5:**
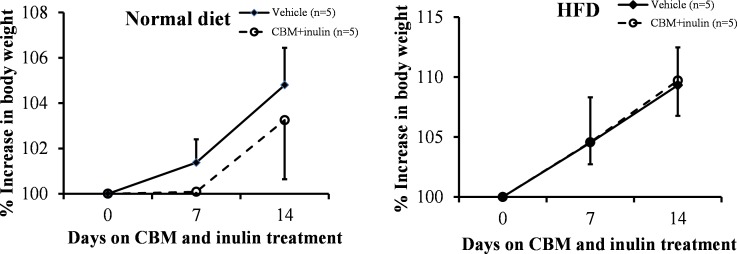
Effects of *Clostridium butyricum* MIYAIRI 588 (CBM) and inulin on body weight (BW). C57BL/6 mice were fed a normal diet or high-fat diet (HFD) supplemented with 3% CBM (wt/wt) and drinking water supplemented with 1% inulin (wt/wt) for 2 weeks. BW was measured weekly. The percentage increase in BW was calculated as: (BW at week W/ BW at week 1) × 100. Bars represent the mean ± SD.

**Fig 6:**
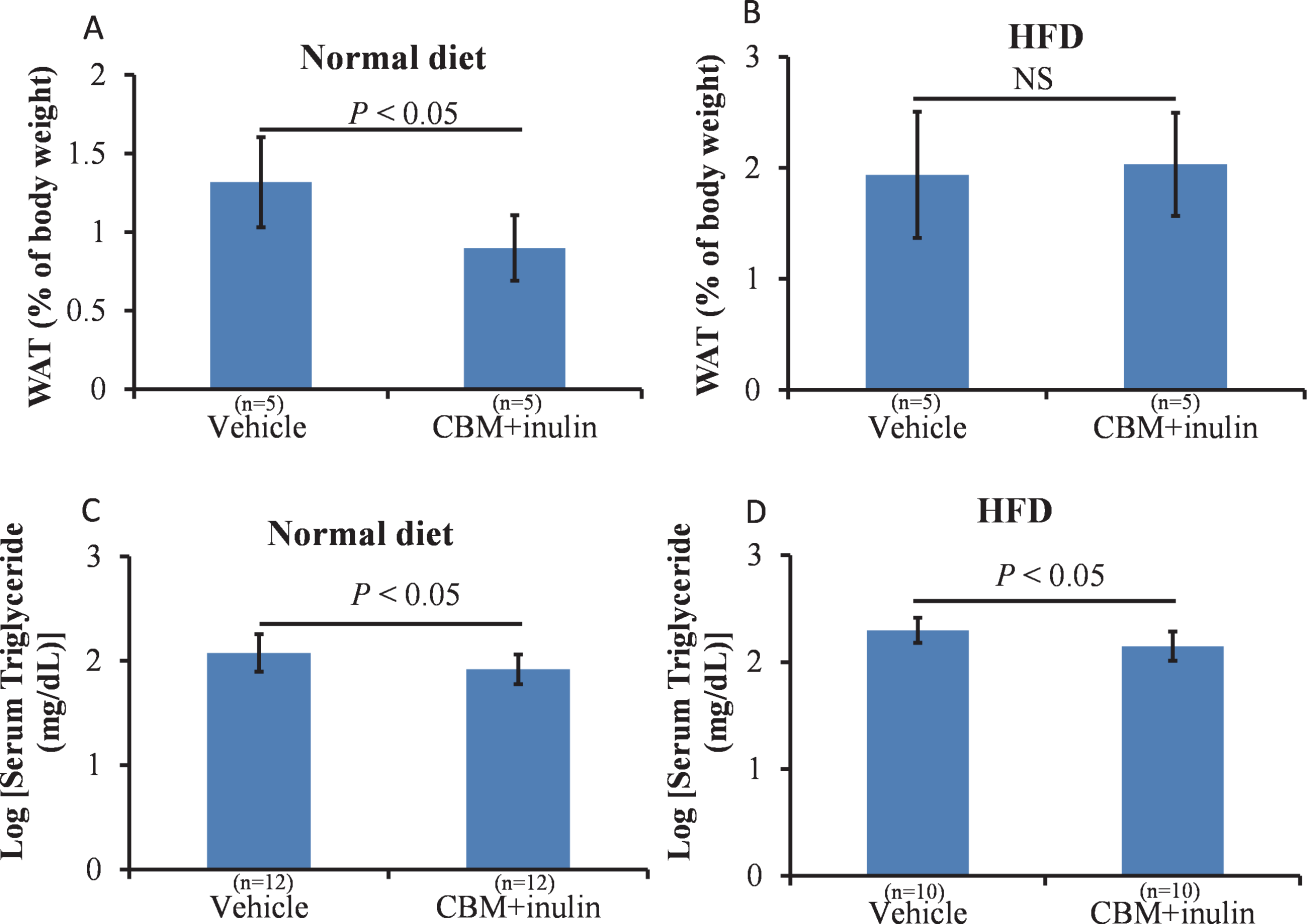
Effects of *Clostridium butyricum* MIYAIRI 588 (CBM) and inulin on adipose tissue and serum triglyceride. C57BL/6 mice were fed a normal diet or high-fat diet (HFD) supplemented with 3% CBM (wt/wt) and drinking water supplemented with 1% inulin (wt/wt) for 2 weeks. **A** and **B**, epididymal white adipose tissue weight was measured. **C** and **D**, Serum triglyceride concentrations were measured. Bars represent the mean ± SD.

**Fig 7:**
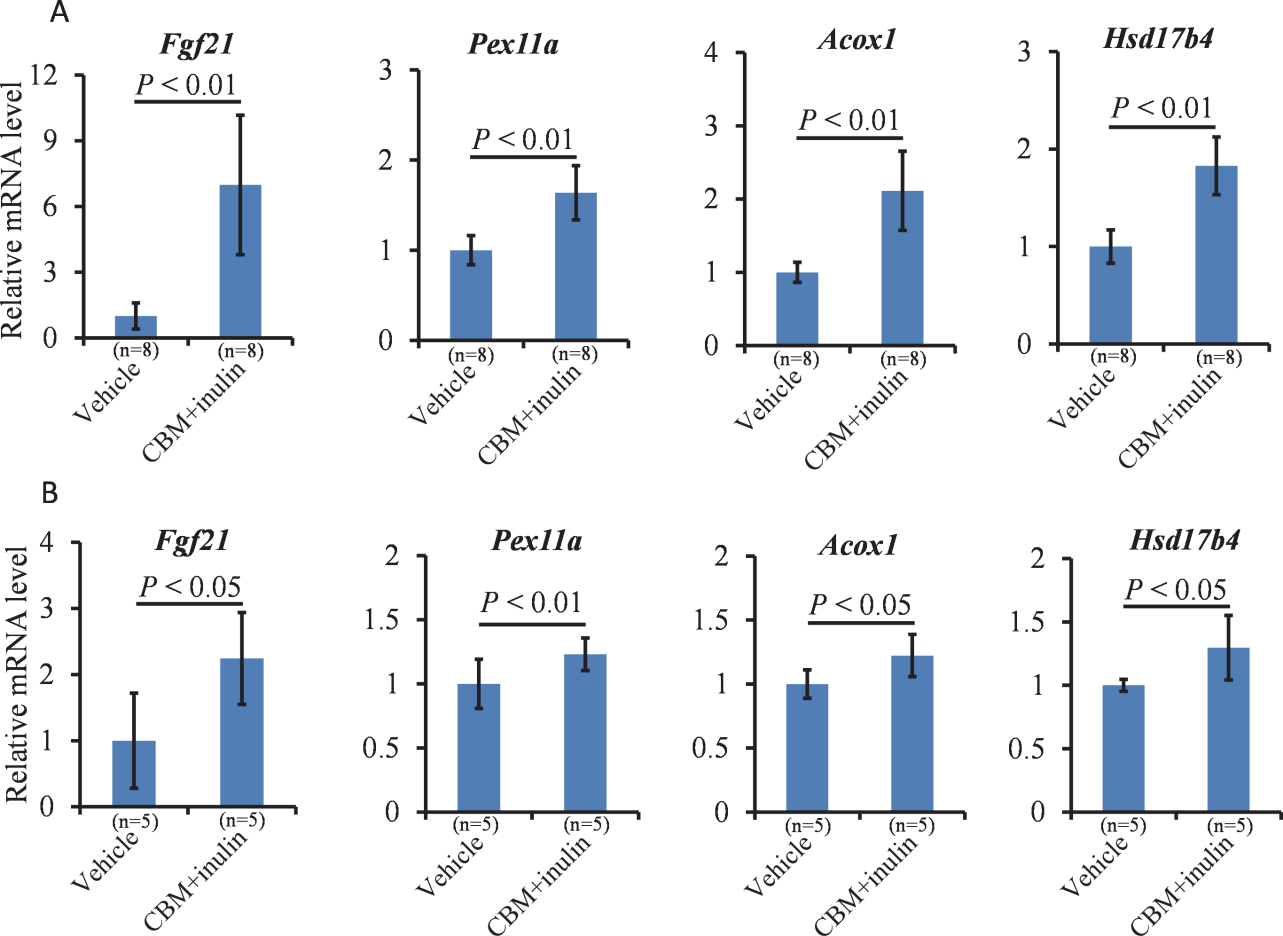
Effects of *Clostridium butyricum* MIYAIRI 588 (CBM) and inulin on mRNA expression in the liver. C57BL/6 mice were fed a normal diet (**A**) or high-fat diet (HFD, **B**) supplemented with 3% CBM (wt/wt) and drinking water supplemented with 1% inulin (wt/wt) for 2 weeks. The mRNA levels of the indicated genes in the livers were measured using real-time reverse transcription-polymerase chain reaction. β-actin was used as an internal control. Bars represent the mean ± SD.

**Fig 8:**
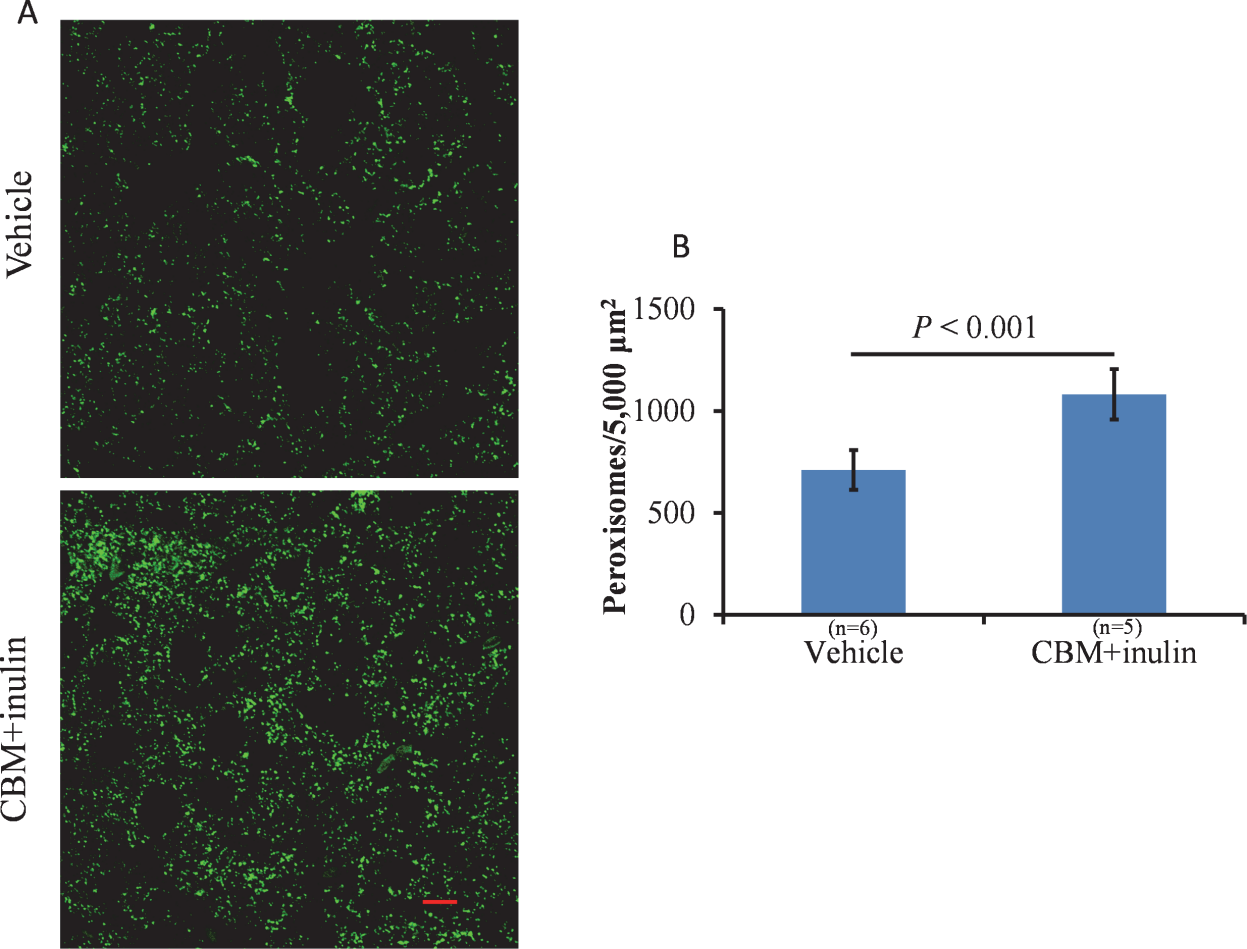
Effects of *Clostridium butyricum* MIYAIRI 588 (CBM) and inulin on peroxisome abundance. C57BL/6 mice were fed a normal diet supplemented with 3% CBM (wt/wt) and drinking water supplemented with 1% inulin (wt/wt) for 2 weeks. **A**, Liver sections were examined using immunofluorescence staining with an antibody to peroxisome membrane protein 70 (Alexa Fluor 488, green channel). Scale bar: 5 μm. The fluorescence emitted by Alexa Fluor 488 was visualized using a confocal laser-scanning microscope. **B**, Peroxisomes were counted using the Particle Analysis package of Image J. Bars represent the mean ± SD.

As described above, microbiota in the colon and distal small intestine can decompose resistant starch, dietary fiber, and low-digestible polysaccharides to produce butyrate [10]. We assessed the effects of CBM or dietary fiber on peroxisome proliferation and lipid metabolism. Our preliminary observations indicated that treatment of CBM only or inulin only was insufficient to reduce serum triglyceride levels and induce *Pex11a* and *Fgf21* expression (S1 Fig.) and to cause peroxisome proliferation (data not shown). We next assessed the effects of CBM plus inulin administration, expecting increased production of butyrate in the colon. Although CBM and inulin treatment did not affect the BW of mice fed a normal diet or an HFD (Fig. 5), the percentage of epididymal white adipose tissue (normalized by BW) was significantly lower in the treated group than that in the control group under the normal diet, but not the HFD (Fig. 6A and 6B, *P* < 0.05). Furthermore, serum triglyceride concentrations in the treated group were significantly lower than those in the control group under both the normal diet and HFD (Fig. 6C and 6D, *P* < 0.05). *Pex11a*, *Fgf21*, *Acox1*, and *Hsd17b4* mRNA levels of the treated group were significantly higher than those of the control group (Fig. 7). The number of peroxisomes in the livers of the treated group was significantly higher than that in the livers of the control group (Fig. 8, *P* < 0.001).

## Discussions

Our previous studies have shown that *Pex11a* deficiency impairs peroxisome elongation and abundance and peroxisomal fatty acid β-oxidation, subsequently contributing to increased lipid accumulation in the liver (steatosis) or kidney (chronic kidney disease) [4, 7]. Pharmacological or other approaches to activate *Pex11a* and/or the peroxisome system may provide an effective therapeutic strategy against these diseases. The present study provides the first evidence that butyrate-producing probiotics plus fiber administration induced peroxisome proliferation and improved lipid metabolism in mice.

Tributyrin, a butyrate acid prodrug, is rapidly absorbed and is chemically stable in plasma. Previous studies showed that tributyrin protects mice against obesity and obesity-associated insulin resistance and dyslipidemia [17]. Consistent with this, we observed increases in *PPARα*, *Pex11a*, and *Fgf21* mRNA levels 6 h after tributyrin injection. *Fgf21*, a metabolic regulator mainly secreted from the liver in response to fasting and starvation under the control of PPARα, plays a critical role in maintaining energy homeostasis and insulin sensitivity [18–22]. Fgf21 administration has been shown to reduce liver and circulating triglyceride levels, adipose tissue, and BW in rodents and nonhuman primates [16]. Although whether or how Fgf21 might affect peroxisome proliferation requires further studies, these effects point to the induction of *Fgf21* as a potential therapeutic strategy against obesity and dyslipidemia.

However, induction of *PPARα* and *Pex11a* was not observed after 24 h of tributyrin administration. A major drawback of tributyrin is that its effects may be attenuated by oral administration.

Next, we observed the effects of 4-PBA, which was supplemented in the drinking water to increase the frequency of its intake and maintain plasma butyrate concentrations. As expected, 4-PBA treatment induced the mRNA expression of *Pex11a* and genes involved in peroxisomal fatty acid β-oxidation and increased peroxisome abundance.

Butyrate is an SCFA produced during the fermentation of fibers and other substrates by resident anaerobic bacteria in the gastrointestinal tract [10]. Elevation of SCFA availability by increasing dietary fiber intake or a diet supplemented with butyrate may prevent the development of metabolic disarrangements and the insulin resistance associated with obesity [17, 23–25]. Previous studies showed that dietary fiber treatment, but not CBM treatment, improved hyperlipidemia in rats fed an HFD [12, 13]. However, in our preliminary experiments, mice were treated by CBM only or inulin only, which was insufficient to reduce serum triglyceride levels and to activate the Fgf21-PPARα pathway (S1 Fig.). Therefore, as a gentler method than tributyrin or 4-PBA administration, we used both CBM and inulin treatment to increase the intrinsic production of butyrate in the colon and butyrate concentrations in the blood [14, 26], and thereby promote butyrate availability in the liver for activation of the Fgf21-PPARα pathway. Interestingly, CBM and inulin treatment induced the mRNA expression of *Pex11a* and *Fgf21* and increased the abundance of peroxisomes, as expected. It also reduced the epididymal white adipose tissue mass and serum triglyceride concentration.

In conclusion, elevation of butyrate availability (directly through administration of butyrate or indirectly via administration of butyrate-producing probiotics plus fiber) induces *PPARα* and *Pex11a* as well as genes involved in peroxisomal fatty acid β-oxidation, increases peroxisome abundance, and ultimately improves lipid metabolism. These results provide a new therapeutic strategy against metabolic syndrome-related diseases.

## Supporting Information

S1 Checklist Completed “The ARRIVE Guideline Checklist” for reporting animal data in this manuscript. (DOCX)

S1 Fig Effects of treatment with *Clostridium butyricum* MIYAIRI 588 (CBM) only or inulin only. C57BL/6 mice were fed a normal diet and drinking water supplemented with 1% inulin (wt/wt) (A), or a high-fat diet (B) supplemented with 3% CBM (wt/wt) for 2 weeks. The mRNA levels of the indicated genes in the livers were measured using real-time reverse transcription-polymerase chain reaction. β-actin was used as an internal control. Bars represent the mean ± SD. NS: not significant.
(TIF)
