# Supplementary figures and images for: Induction of Peroxisomes by Butyrate-Producing Probiotics
Source: PLoS One. 2015 Feb 6;10(2):e0117851. doi: 10.1371/journal.pone.0117851 (PMC4320100; doi:10.1371/journal.pone.0117851)

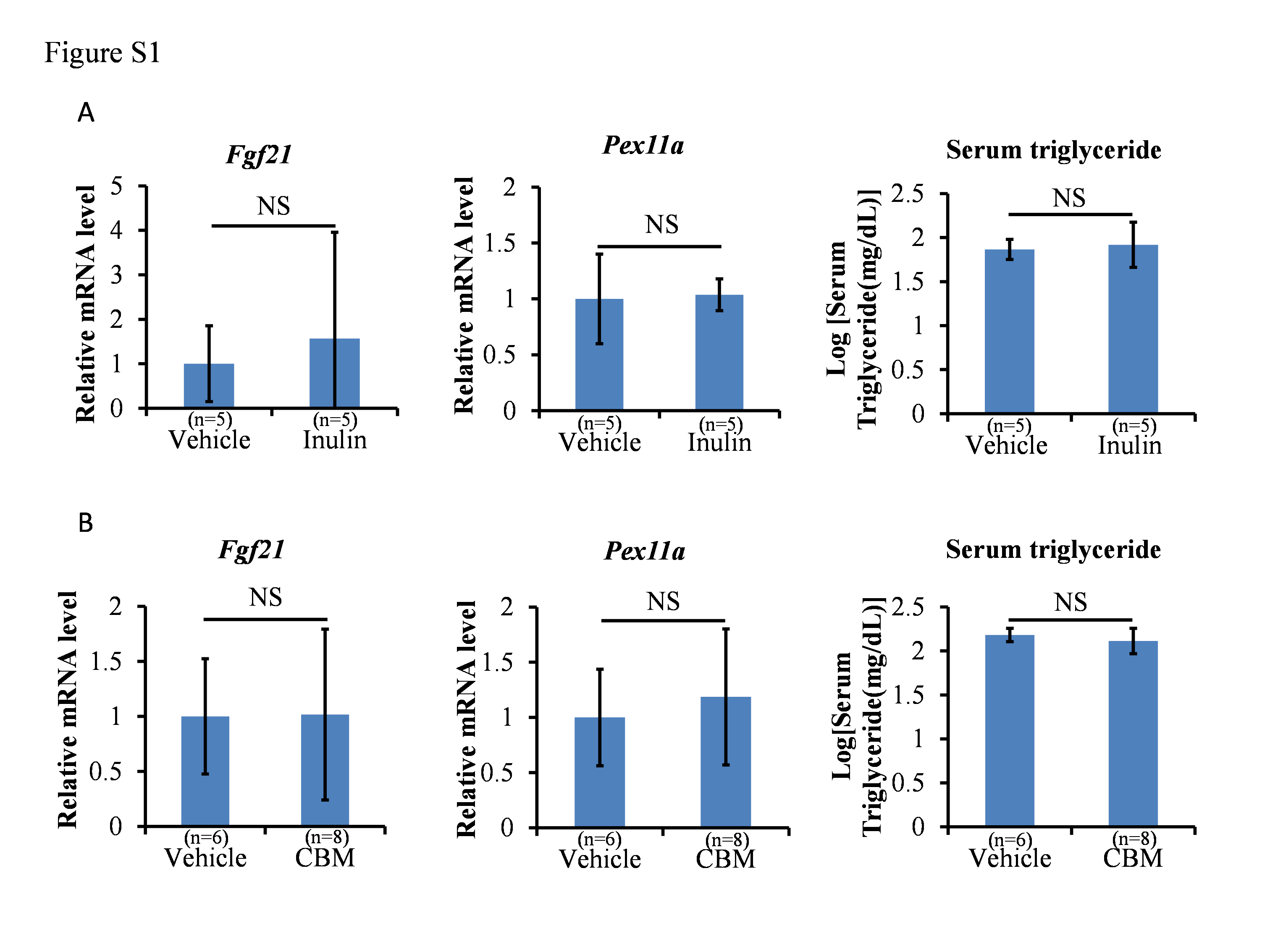

Supplement: S1 Fig — C57BL/6 mice were fed a normal diet and drinking water supplemented with 1% inulin (wt/wt) (A), or a high-fat diet (B) supplemented with 3% CBM (wt/wt) for 2 weeks. The mRNA levels of the indicated genes in the livers were measured using real-time reverse transcription-polymerase chain reaction. β-actin was used as an internal control. Bars represent the mean ± SD. NS: not significant. (TIF) [file pone.0117851.s002.tif]
